# Role of *CES1* and *ABCB1* Genetic Polymorphisms on Functional Response to Dabigatran in Patients with Atrial Fibrillation

**DOI:** 10.3390/jcm13092545

**Published:** 2024-04-26

**Authors:** Luca Cumitini, Giulia Renda, Mara Giordano, Roberta Rolla, Tarek Shail, Sara Sacchetti, Lorena Iezzi, Luca Giacomini, Valentina Zanotti, Raffaella Auciello, Ilaria Angilletta, Melissa Foglietta, Mirco Zucchelli, Ivana Antonucci, Liborio Stuppia, Sabina Gallina, Umberto Dianzani, Giuseppe Patti

**Affiliations:** 1 Department of Translational Medicine, University of Eastern Piedmont, 28100 Novara, Italy; lucacumitini@gmail.com (L.C.); tarek.shail@gmail.com (T.S.); 2 Division of Cardiology, AOU Maggiore della Carità, 28100 Novara, Italy; 3 Institute of Cardiology, Department of Neuroscience, Imaging and Clinical Sciences, G. d’Annunzio University of Chieti-Pescara, 66100 Chieti, Italy; giulia.renda@unich.it (G.R.); lorenaie@outlook.com (L.I.); iangilletta3@gmail.com (I.A.); melissa.foglietta11@gmail.com (M.F.); sabina.gallina@unich.it (S.G.); 4 Center for Advanced Studies and Technology (CAST), G. d’Annunzio University of Chieti-Pescara, 66100 Chieti, Italy; mirco.zucchelli@unich.it (M.Z.); i.antonucci@unich.it (I.A.); liborio.stuppia@unich.it (L.S.); 5 Department of Health Sciences, University of Eastern Piedmont, 28100 Novara, Italy; mara.giordano@med.uniupo.it (M.G.); roberta.rolla@med.uniupo.it (R.R.); sara.sacchetti@uniupo.it (S.S.); 20023552@studenti.uniupo.it (L.G.); 10032928@studenti.uniupo.it (V.Z.); umberto.dianzani@med.uniupo.it (U.D.); 6 Clinical Biochemistry, AOU Maggiore della Carità, 28100 Novara, Italy; 7 Department of Clinical Pathology, Renzetti Hospital, Lanciano (Chieti), 66034 Lanciano, Italy; raffaella.auciello@asl2abruzzo.it; 8 Department of Innovative Technologies in Medicine, G. d’Annunzio University of Chieti-Pescara, 66100 Chieti, Italy; 9 Department of Psychological, Health and Territory Sciences, G. d’Annunzio University of Chieti-Pescara, 66100 Chieti, Italy

**Keywords:** oral anticoagulants, dabigatran, genetics, polymorphisms, diluted thrombin time

## Abstract

**Background**: Dabigatran etexilate is a pro-drug hydrolyzed into dabigatran by carboxylesterases (CES) and is a substrate of the P-Glycoprotein encoded by the adenosine-triphosphate-binding cassette sub-family B member (ABCB)1 genes. We evaluated the functional response to dabigatran according to different CES1 and ABCB1 single-nucleotide polymorphisms (SNPs) in patients with atrial fibrillation (AF). **Methods**: A total of 100 consecutive patients with AF taking dabigatran were enrolled by two Italian centers. A venous blood sample was drawn for genetic determinations, as well as a measurement of the diluted thrombin time (dTT) and drug plasma concentrations, at the trough and peak. The main objective was the relationship between the dTT values and *CES1 rs2244613*, *CES1 rs8192935* and *ABCB1 rs4148738* SNP while on two different dabigatran doses (110 and 150 mg BID). **Results**: A total of 43 patients were on a 110 mg dabigatran dose and 57 on 150 mg. The DTT values at the trough and at peak were not different among patients with different *CES1 rs2244613* and *CES1 rs8192935* genotypes, regardless of the dabigatran dose. In patients on 150 mg dabigatran, the dTT values at the trough were 77 (44–111) ng/mL in patients with the *ABCB1 rs4148738* heterozygous *CT* genotype vs. 127 (85–147) ng/mL in the wild-type CC genotype vs. 110 (47–159) ng/mL in the mutant trait TT genotype (*p* = 0.048). In patients with the *ABCB1 rs4148738 CT* genotype, OR for having dTT values at a trough below the median was 3.21, 95% CI 1.04–9.88 (*p* = 0.042). **Conclusions**: *ABCB1 rs4148738 CT* heterozygous is associated with the reduced anticoagulant activity of dabigatran at the trough in patients receiving the higher dose regimen.

## 1. Introduction

Atrial fibrillation (AF) is the most common cardiac arrhythmia worldwide and the leading cause of ischemic stroke [1]. AF treatment is largely focused on preventing thromboembolic events through chronic anticoagulant therapy [2]. In this regard, direct oral anticoagulants (DOACs) currently represent the guideline-recommended first-line treatment. These drugs have proven to be at least equally effective as warfarin in preventing AF-related stroke, ref. [3] with greater safety in terms of major bleeding reduction, especially intracranial hemorrhages. The direct thrombin inhibitor dabigatran is a representative DOAC widely used in patients with AF or venous thromboembolism [4]. Dabigatran etexilate is a pro-drug which is rapidly hydrolyzed into dabigatran by means of esterases, such as carboxylesterase 1 (*CES1*) and *CES2*. *CES1* catalyzes the conversion of dabigatran etexilate to dabigatran in the liver, while intestinal *CES2* plays a compensatory role when *CES1* is inhibited [5]. Dabigatran etexilate, but not dabigatran, is a substrate of the P-Glycoprotein (P-gp), encoded by the adenosine-triphosphate (ATP)-binding cassette sub-family B member (*ABCB*)1 genes. Potent P-gp inhibitors increase the bioavailability of dabigatran from 12% to 23%, whereas P-gp stimulators reduce the plasmatic concentrations of the drug [6].

Dabigatran has a predictable pharmacokinetic profile, allowing for a fixed-dose regimen. However, inter-individual variability in the pharmacokinetic and pharmacodynamic response to dabigatran has recently been reported [7]. In particular, it was demonstrated that single-nucleotide polymorphisms (SNPs) in *CES1* and *ABCB1* genes lead to inter-individual differences influencing drug metabolism and bioavailability and, as a consequence, affecting thrombotic protection and bleeding risk [7,8]. Notably, data from available studies on the association between *CES1/ABCB1* SNPs, drug concentrations and event risk are controversial. For instance, the presence of the *CES1 rs2244613* G allele was associated with reduced dabigatran concentrations at the trough compared to the T allele and with a lower bleeding risk [7]. However, Shi et al. [9] observed that this gene locus may be unrelated to the dabigatran concentrations and consequent clinical outcome.

Routine coagulation assays, such as the activated partial thromboplastin time and prothrombin time, resulted inadequacy for accurately measuring the anticoagulant effects of dabigatran, whereas in in vitro studies of the thrombin time and ecarin clotting time showed a linear and steep dose–response relationship [10]. However, the thrombin time is too sensitive, with the excessive prolongation of the measurement time, while the ecarin clotting time is not widely available or used [10]. In recent years, a commercial assay for measuring plasmatic dabigatran has become available (Hemoclot^®^ Thrombin Inhibitor). It is based on the thrombin time, in which a plasmatic sample is diluted with saline solution and normal pool plasma before the initiation of coagulation with thrombin. Such a diluted thrombin time (dTT) yields shorter coagulation times [11] and is applicable to different coagulation analyzers. The International Society for Thrombosis and Hemostasis (ISTH) currently recommends that a dabigatran-calibrated dTT can be used to estimate the plasma levels and antithrombin effects of the drug.

To date, no study has evaluated the association of *CES1* and *ABCB1* SNPs with the anticoagulant activity of dabigatran in patients with AF. The main contribution of the present investigation is to evaluate the relationship between the aforementioned genetic polymorphisms and the functional response to dabigatran, as measured by dTT, in a selected population of Caucasian patients.

## 2. Methods

### 2.1. Ethics

This study was designed following Declaration of Helsinki, Good Clinical Practice (GCP) guidelines and other related guiding principles. The protocol was approved by the Ethics Committee of the two involved tertiary centers: “Maggiore della Carità” Hospital, Novara, Italy (Coordinating center, Ethics approval number: CE 129/21, 29 October 2021) and “SS. Annunziata” Hospital, Chieti, Italy. Signed informed consent was obtained from all subjects included in the investigation. According to the design, all results were used only for scientific purposes without uncovering personal identifiers.

### 2.2. Study Population

We performed a prospective, observational study in consecutive patients with AF. Eligible patients were those taking dabigatran according to the registered clinical indications and dosages. A total of 100 patients were enrolled at the Cardiology Division of “Maggiore della Carità” Hospital of Novara, Italy (*n* = 56) and at Cardiology Division of “SS. Annunziata” Hospital of Chieti, Italy (*n* = 44) from December 2021 to December 2022. Specific inclusion criteria were: age ≥18 years; any type of AF; CHA_2_DS_2_-VASc score ≥1; dabigatran treatment for at least two weeks (either 110 mg or 150 mg twice daily); and ability to provide written informed consent. Exclusion criteria were: CHA_2_DS_2_-VASc score of 0; moderate-to-severe mitral stenosis; mechanical prosthetic heart valve; recent (<1 month) electrical cardioversion or ablation of AF; left ventricular ejection fraction <40%; myocardial infarction or percutaneous coronary intervention <6 months; cardiac or non-cardiac surgery <6 months; creatinine clearance <50 mL/min; concomitant antiplatelet therapy; active ongoing bleeding; platelet count <50,000/μL; any stroke <1 month; fibrinolytic therapy <10 days; or hemoglobin level <9 g/dL. From each patient, we obtained a venous blood sample drawn at 8:00 a.m. for both genetic determinations and measurement of coagulative parameters at trough of plasma concentration (approximately 12 h after the last drug assumption). The second venous blood sample was taken 2–3 h later than the dabigatran morning dose for the assessment of coagulative parameters at peak of plasma concentration, according to the drug pharmacokinetic profile [12].

### 2.3. Determination of Blood Coagulation Parameters and Plasma Concentration of Dabigatran

Blood samples were collected by a venipuncture into 3 mL tubes containing 1:9 sodium citrate (for dabigatran plasma concentration measurement) and 3 mL tubes with K3 EDTA (for coagulation dTT test). All patients were fasting for at least 2 h at the time of blood sampling. All participants were taking dabigatran from a single, unique manufacturer. Blood samples were then centrifuged for 15 min at 3000× *g* and, subsequently, 0.5 mL of the upper layer of platelet–poor plasma was aspirated, frozen and stored at −80 °C until analysis was performed. Dabigatran plasma concentrations were measured by high-performance liquid chromatography/electrospray ionization tandem mass spectrometry (HPLC-ESIMS-MS) method, which has been shown to be linear over a range of dabigatran plasma concentrations of 1–500 ng/mL. For free dabigatran quantification, 200 μL aliquots of unknown plasma samples, calibrators and quality controls (3, 75 and 375 ng/mL prepared in pooled drug-free human plasma) were transferred to a 1.5 mL polypropylene tube and 600 μL of methanol containing internal Standard [^13^C6]-dabigatran 50 ng/mL were added. The sample was vortex-mixed and subsequently centrifuged for 5 min at 12,000 RCF (relative centrifugal force). The supernatant liquids were transferred to new tubes and the extracted samples were evaporated to dryness in a vacuum concentrator. The dry residues were reconstituted in 200 μL of H_2_O and transferred to vials for the analysis. A total of 25 μL were injected into the liquid chromatography coupled to tandem mass spectrometry (LC-MS/MS) system, consisting of an UPLC Acquity I class coupled via electrospray to a triple quadrupole mass spectrometer Xevo TDQ (Waters Corp, Wood Dale, IL, USA). The chromatographic separation was carried out using a CORTECS UPLC C18 1.6 μm 2.1 × 150 mm column at a temperature of 40 °C. The mobile phase consisted of a 25% acetonitrile–75% water mixture acidified (0.1%) with formic acid (isocratic condition, flow rate 0.1 mL/min, run time 5 min). The system was operated in positive electrospray ionization (ESI+) mode. Dabigatran and internal standard [^13^C6]–dabigatran were monitored by multiple reaction monitoring (MRM) method (transitions m/z 478.2 > 295.1 for IS). MassLynx V4.2 software (Waters Corp.) for instrument control, analysis, data acquisition and processing was used.

Coagulation dTT tests on automated analyzers were performed. The HEMOCLOT test (HYPHEN BioMed, Paris, France) was used to measure the pharmacodynamic effects of dabigatran by diluting the test sample with 0.15 mol/L of NaCl and adding a constant amount of α-thrombin to initiate coagulation. The dilution of the test sample avoids the oversensitivity of the assay response associated with the conventional thrombin time test. The diluted plasma (1:8) was mixed with normal human plasma provided in the HEMOCLOT test kit (HYPHEN BioMed, Paris, France). Coagulation was then initiated by the addition of a constant amount of highly purified human α-thrombin. Clotting times using standard magnetic ball coagulometers were determined. Assay validation and data evaluation procedures followed recent recommendations. The HEMOCLOT test provides a dabigatran concentration as a result, expressed in ng/mL. This measurement is obtained by correlation between the dTT, measured with the coagulometer after the addition of α-thrombin, and known standard dilutions of dabigatran, supplied in the HEMOCLOT analysis kit. Therefore, dTT is not expressed as a measurement of time, but as a concentration, even if it is calculated indirectly. To differentiate between these indirect concentrations expressing dTT and direct concentrations measured by the LC-MS/MS method, the former have always been expressed, in any case, as dTT in ng/mL [13].

### 2.4. Genotyping

Venous blood samples were collected using the Vacuette vacuum system (Greiner Bio-One, Kremsmünster, Austria) in test tubes containing K3 EDTA, stored at the temperature of +4 °C and extracted <1 month after collection. DNA extraction was performed using the Maxwell Whole Blood DNA kit on Maxwell^®^ RSC instrument (Promega, Hollow Road Madison, WI, USA). After 5 min at room temperature, approximately 400 μL of each blood sample was transferred from the starting tube to well n. 1 of each cartridge. Blood was drawn in four times (100 μL for each draw) to avoid transfer of clots and 60 μL of elution buffer was added to the bottom of each elution tube. The Maxwell automated system was then started to progressively transfer the blood sample into the cartridge in a progressive manner. The DNA obtained was stored in 0.5 mL Eppendorf tubes at a temperature of −20 °C.

Polymorphisms *CES1 rs2244613*, *CES1 rs8192935* and *ABCB1 rs4148738* were included in the study as potential genetic determinants of the pharmacodynamic response to dabigatran. Allelic variants were determined using a Real-Time Polymerase Chain Reaction (RT-PCR) protocol with commercial TaqMan^®^ SNP Genotyping Assays kits (Thermo Fisher Scientific, Waltham, MA, USA) for all individual polymorphisms. Each TaqMan predesigned SNP Genotyping Assay contains two differentially allele-specific TaqMan probes labeled with VIC^®^ and FAM^®^ fluorescent detectors. In addition, each probe contains two unlabeled PCR primers, one forward and one reverse, that amplify specifically the allele of interest. The VIC-labeled probe was coupled to the wild-type allele, while the FAM-labeled probe was coupled to the mutant allele.

- AGATCAAAAAGTGACCA CAGCCCC[G/T]GGTGAGCGATGCAGCTTCTCCAGCC

*CES1* nucleotide sequence, SNP ID: rs2244613 [VIC/FAM]. Type of polymorphism: guanine/thymine transversion.

- TACAAACAATATATTACATCATAAT[A/G]CTTTACCATCTAAAATACTGATAGT

*CES1* nucleotide sequence, SNP ID: rs8192935 [VIC/FAM]. Type of polymorphism: adenine/guanine transition.

- AGGGTTGAGGGGAGGAACTAAAACC[C/T]GTCAGCCAAAAACAGGTCAGCTAGT

*ABCB1* nucleotide sequence, SNP ID: rs4148738 [VIC/FAM]. Type of polymorphism: cytosine/thymine transition.

The RT-PCR program consisted of a preliminary denaturation at 95 °C for 2 min, followed by 39 cycles of 15 s each, denaturation at 95 °C and annealing at 56 °C for 1 min. At the end of the above program, allelic discrimination was measured by evaluating change in fluorescence of the fluorophore associated with the probes: a substantial increase only in VIC fluorescence was associated with the homozygous genotype for the wild-type allele, a substantial increase in the FAM fluorescence only was associated with the homozygous genotype for the mutant allele and a substantial increase in both fluorescences was associated with heterozygosis.

### 2.5. Endpoints

Main endpoints were:

- Evaluation of peak and trough dTT values based on *CES1 SNP rs2244613*, using both doses of dabigatran (110 mg and 150 mg BID);
- Evaluation of peak and trough dTT values based on *CES1 SNP rs8192935*, using both doses of dabigatran;
- Evaluation of peak and trough dTT values based on *ABCB1 SNP rs4148738*, using both doses of dabigatran.

### 2.6. Statistical Analysis

Continuous variables are reported as median and quartile 1 to quartile 3 (interquartile range [IQR]) in the case of skewed distributions, as in the case of dTT values and drug concentrations, or as mean and standard deviation (SD) for data with normal distribution. Categorical variables were listed as absolute numbers and percentages. The presence of normal distribution was verified by Shapiro–Wilks test. The Mann–Whitney test was used for statistical comparison of continuous, non-normally distributed variables and the Student *t*-test for normally distributed variables. Comparisons between different genetic polymorphisms were performed by Kruskal–Wallis analysis. Spearman’s rank correlation was applied to evaluate the relationship between dabigatran concentration and dTT, both at trough and at peak. The effect of each genetic variant on dTT value was analyzed initially by a univariate linear regression analysis considering dTT value as continuous. Then, we applied a conditional logistic regression model using dTT value as discrete. The null hypothesis of no association was tested with the likelihood ratio test. The effect of each genetic variant for having a dTT value at trough and at peak below the median was expressed as Odds Ratio (OR) and 95% confidence interval (CI). Statistical significance was indicated by a *p* value < 0.05. The statistical software STATA 18.0 (StataCorp., LP, College Station, TX, USA) was used to generate statistical models and analyze data.

## 3. Results

**Figure 1:**
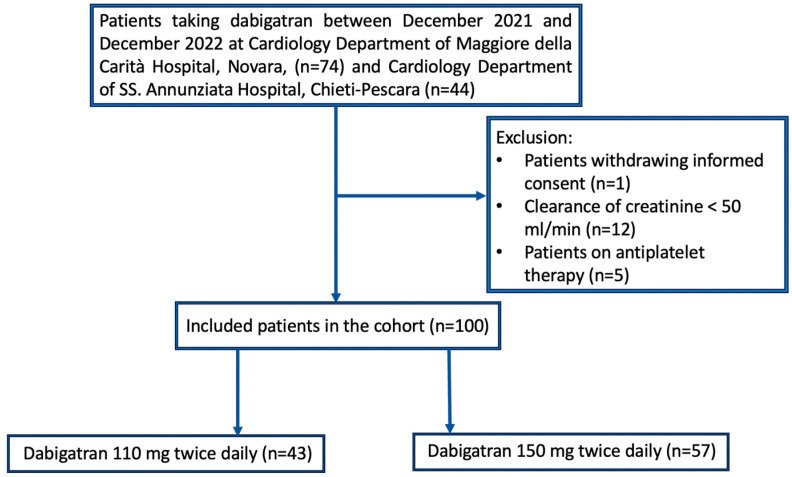
Flow diagram indicating how the final study sample was obtained.

**Figure 2:**
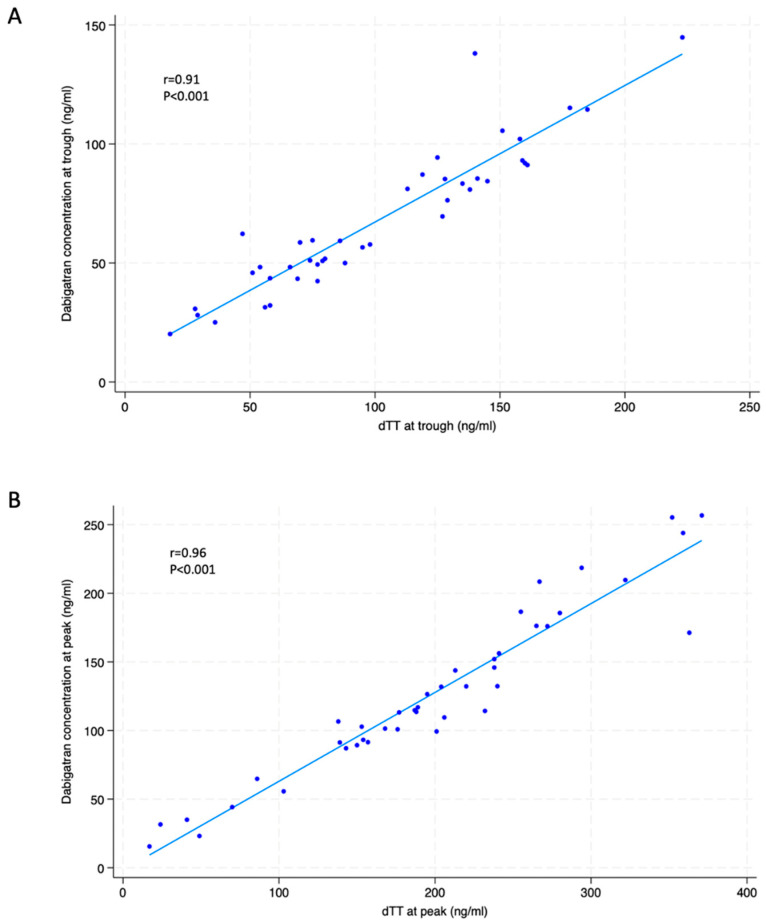
Correlation between dabigatran plasma concentration and dTT at trough (**A**) and at peak (**B**). dTT= diluted thrombin time.

Figure 1 indicates how the final study sample of 100 patients was obtained. All participants were Caucasian and Italian. Table 1 shows the demographic and clinical characteristics of the included population. The median age was 77 years, with 63 patients (63%) being of male gender. Regarding the type of AF, 52 patients (52%) had paroxysmal, 22 (22%) persistent and 26 (26%) permanent AF. A total of 43 patients (43%) were on a 110 mg BID dabigatran dose and 57 (57%) on a 150 mg BID dabigatran regimen. Patients on 110 mg dabigatran were older (82 vs. 70 years, *p* < 0.001), with a lower body weight (74 vs. 83 Kg, *p* = 0.002) and reduced creatinine clearance (60 vs. 81 mL/min, *p* < 0.001) than those taking 150 mg dabigatran. In the overall population, the dabigatran plasma concentration was 59 (48–87) ng/mL at the trough and was 115 (91–171) ng/mL at the peak; dTT was 90 (51–132) ng/mL at the trough and 176 (101–232) ng/mL at the peak (Table 2). A significant correlation was observed between the drug concentrations and dTT at the trough (r = 0.91; *p* < 0.001) and dTT at the peak (r = 0.96; *p* < 0.001) (Figure 2). Given this high-degree correlation, only data on the association between genetic polymorphisms and the dTT at the trough and peak (main study endpoint) are specifically reported in the text, whereas results on the association between the genetic polymorphisms and plasma concentration of dabigatran are indicated only in the Supplementary Tables.

### 3.1. CES1 rs2244613 Polymorphism

A total of 5 patients had the homozygous GG genotype (wild-type) of *CES1 rs2244613*, while 35 patients had the heterozygous GT genotype and 60 patients had the homozygous TT genotype (mutant trait) (Table 3). The prevalence of different *CES1 rs2244613* genotypes according to different dabigatran doses is indicated in Table 3.

**Figure 3:**
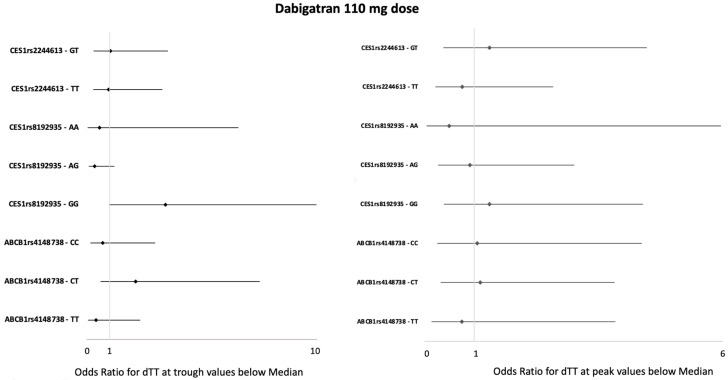
Forest plot of the effect of genetic variants on the risk of having a below-the-median dTT value at trough (**left-side forest plot**) and at peak (**right-side forest plot**) while on dabigatran 110 mg dose. The genetic variants evaluated are listed on the left and are grouped according to the gene where they are located. The plot shows for each genetic variant the specific effect on dTT [expressed as ORs and 95% CIs (horizontal lines)] against both the alternative genotypes at the same locus. The homozygosis not shown (*CES1 rs2244613 GG*) was not present in our population. The data were analyzed by a conditional logistic regression model by using dTT as the discrete variable. The null hypothesis of no association was tested with the likelihood ratio test. The vertical line represents no effect. The overlap of the CI with this line indicates that the effect size does not deviate significantly from no effect. dTT = diluted thrombin time.

**Figure 4:**
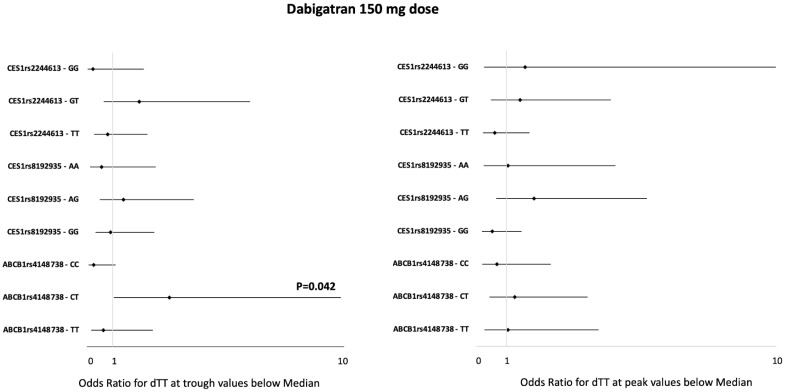
Forest plot of the effect of genetic variants on the risk of having a below-the-median dTT value at trough (**left-side forest plot)** and at peak (**right-side forest plot)** while on dabigatran 150 mg dose. The genetic variants evaluated are listed on the left and are grouped according to the gene where they are located. The plot shows for each genetic variant the specific effect on dTT [expressed as ORs and 95% CIs (horizontal lines)] against both the alternative genotypes at the same locus. The data were analyzed by a conditional logistic regression model by using dTT as the discrete variable. The null hypothesis of no association was tested with the likelihood ratio test. The vertical line represents no effect. The overlap of the CI with this line indicates that the effect size does not deviate significantly from no effect. dTT = diluted thrombin time.

In patients taking both dabigatran doses, ORs for having dTT values at the trough and peak below the median, according to different *CES1 rs2244613* polymorphisms, were not significant (Figure 3 and Figure 4). dTT values at the trough and peak did not differ between patients with different *CES1 rs2244613* genotypes, regardless of the dabigatran dose (Supplementary Table S1).

### 3.2. CES1 rs8192935 Polymorphism

*CES1 rs8192935* homozygous AA genotype (wild-type) was found to be present in 11 patients, the heterozygous AG genotype in 43 patients and the homozygous GG genotype (mutant trait) in 46 patients (Table 3). The prevalence of different *CES1 rs8192935* genotypes according to different dabigatran doses is indicated in Table 3.

In patients taking both dabigatran doses, ORs for having dTT values at the trough and at peak below the median according to different *CES1 rs8192935* polymorphisms were not significant (Figure 3 and Figure 4). Patients with the *CES1 rs8192935* GG genotype receiving the 110 mg dabigatran dose showed a propensity to have lower dTT values at the trough (OR 3.42, 95% CI 0.97–12.09; *p* = 0.06). dTT values at the trough and peak were not statistically different between patients with different *CES1 rs8192935* genotypes, regardless of the dabigatran dose (Supplementary Table S2). We also performed an analysis on combinations of mutations: patients with the TT mutant genotype for CES1 rs2244613 and the GG mutant genotype for CES1 rs8192935 and patients with the compound heterozygosity of CES1 rs2244613 and CES1 rs8192935. ORs for having dTT values at the trough and peak below the median in patients with both the TT mutant genotypes for *CES1 rs2244613* and the GG mutant genotype for *CES1 rs8192935* (44 patients) were not significant, regardless of the dabigatran dose. ORs for having dTT values at the trough and peak below the median in patients with the compound heterozygosity of *CES1 rs2244613*—GT and *CES1 rs8192935*—AG (27 patients) were not significant, regardless of the dabigatran dose.

### 3.3. ABCB1 rs4148738 Polymorphism

The genotype frequencies of *ABCB1 rs4148738* were the following: the homozygous CC genotype (wild-type) in 22 patients, the heterozygous CT genotype in 61 patients and the homozygous TT genotype (mutant trait) in 17 patients (Table 3). The prevalence of different *ABCB1 rs4148738* genotypes according to different dabigatran doses is indicated in Table 3. A slight departure from the Hardy–Weingberg equilibrium (*p* = 0.02) was observed, which might be attributable to the small sample size or population substructure.

ORs for having dTT values at the trough and peak below the median according to different *ABCB1 rs4148738* polymorphisms were not significant in patients on 110 mg dabigatran (Figure 3). The analysis on patients receiving the 150 mg dabigatran regimen showed a significantly higher probability for dTT values at the trough below the median in patients with the *ABCB1 rs4148738 CT* genotype (OR 3.21, 95% CI 1.04–9.88; *p* = 0.042) (Figure 4). Accordingly, here, the dTT values at the trough were 77 (44–111) ng/mL vs. 127 (85–147) ng/mL in wild-type homozygous patients with the CC genotype vs. 110 (47–159) ng/mL in homozygous carriers of the mutant trait TT genotype (*p* = 0.048) (Table 4). This reduction in dTT values in patients with the *ABCB1 rs4148738 CT* genotype was paralleled by a decreased drug concentration at the trough: 49 (32–59) ng/mL vs. 85 (68–119) ng/mL in the wild-type homozygous CC genotype vs. 78 (62–93) ng/mL in the mutant trait TT genotype (*p* = 0.02).

## 4. Discussion

In the present study, we evaluated the association between *CES1* and *ABCB1* SNPs and dTT values during dabigatran therapy in a real-world cohort of Caucasian patients with AF. In this clinical setting, previous studies have correlated genetic variants at the *CES1* and *ABCB1* locus with dabigatran concentrations. To the best of our knowledge, this is the first study exploring a possible interaction between these genetic polymorphisms and the anticoagulant effect of this drug. Notably, we specifically investigated such an interaction according to two different dabigatran dose regimens.

In general, genetic polymorphisms of drug-metabolizing enzymes, transporters and receptors have been identified as major causes of inter-individual variability in the response to various agents [14]. In particular, recognizing inter-individual variations in the efficacy of anticoagulant drugs due to a genotype effect has clinical relevance, as it may help predict which patients are at a higher risk of increased efficacy (e.g., of bleeding complications) or treatment failure (e.g., of thrombotic events). Dabigatran is given without routine coagulation monitoring. However, current recommendations indicate that a dabigatran-calibrated dTT can be used in selected cases to estimate drug levels with the antithrombin effect, such as in patients at the extreme of body weight; with concerns related to low compliance or drug interactions; with possible drug overdosing or accumulation in the case of spontaneous bleeding, liver insufficiency or renal failure; candidates for urgent surgery; or those with ischemic stroke suitable for lytic treatment [11,15].

Our investigation showed that the distribution of the allelic frequencies of SNPs in the *CES1* and *ABCB1* genes in the population was slightly similar to that reported in previous studies, in patients with and without AF [5,16,17].

The relationship between *CES1* polymorphisms and dabigatran concentrations at the peak was previously investigated by Liu et al. [5] in healthy Chinese subjects taking a single oral dose of dabigatran. Here, *CES1 SNP rs2244613* was not associated with drug concentrations, whereas the G allele of *CES1 rs8192935* was associated with increased concentrations. In the RE-LY trial, each minor allele (A) of the *CES1 SNP rs8192935* was associated with a 12% decrease in peak concentrations, but this variability of both peak and trough levels according to *CES1 rs8192935* genetic variants had no clinical impact on bleeding or ischemic events [7]. Notably, each minor allele (C) of the *CES1 SNP rs2244613* was related to lower trough concentrations (15% decrease per allele) and a reduced risk of any bleeding [7]. Finally, Dimatteo et al. [18] showed that *CES1 SNP rs8192935* significantly influenced dabigatran concentrations at the trough, rather than at the peak, and carriers of the T allele had reduced drug concentrations compared to CC genotype carriers [18]. The linkage disequilibrium of the *CES1* SNP with unknown functional variants might also contribute to alterations in the plasma levels of the drug. Indeed, *CES1* primarily converts dabigatran etexilate into its active metabolite in the liver, but a part of dabigatran etexilate can also be metabolized through intestinal *CES2*. Such flexibility in the dabigatran metabolism suggests that an impairment in *CES1* activity is compensated by the activation of the intestinal *CES2* [16,18]. This might also explain the divergence between the conclusions of different reports. In the present study, the correlation between drug concentrations and dTT values was very high and we found no correlation between *CES1 SNP rs2244613* or *CES1 SNP rs8192935* and peak or trough dTT values. Thus, according to our data, the catalytic conversion of the pro-drug dabigatran etexilate to its active form is independent of *CES1 SNP rs2244613* or *CES1 SNP rs8192935*. Consequently, the anticoagulant effect, reflecting active drug concentrations, was similar regardless of those *CES1* variants. Interestingly, our findings might support the aforementioned theory of the compensatory activation of intestinal *CES2*.

According to previous studies, *ABCB1* polymorphisms may alter DOAC exposure, particularly for dabigatran [5,16]. *ABCB1* SNPs are loss-of-function alleles which theoretically result in lower intestinal dabigatran efflux and higher plasma drug levels. In RE-LY, each C allele of *ABCB1 rs4148738* was associated with a 12% decrease in the peak concentration of dabigatran, although this had no impact on bleeding risk reduction [7]. Anna et al. [19] showed in a retrospective study that carriers of the *ABCB1 rs4148738* polymorphism tend to have higher plasma levels of dabigatran, leading to a significantly increased bleeding risk. However, Liu et al. [5] reported that *ABCB1 SNP rs4148738* was not linked to variability in dabigatran concentrations at the peak in AF patients and Sychev et al. [16] showed that there was no influence of the *ABCB1 rs4148738* polymorphism on the dabigatran peak and plasma concentrations in 60 patients after knee surgery who received 220 mg dabigatran etexilate once daily for 1 month for the prevention of deep vein thrombosis. Therefore, in some studies, the *ABCB1* minor allele carriers had higher DOAC plasma concentrations without statistical significance [16] or showed no association with their levels [20,21].

In our study we found that the *ABCB1 rs4148738 CT* genotype has a relationship with the dTT values; this was observed only in patients taking the higher dabigatran regimen and only for dTT at the trough. Thus, based on our findings, the activity and expression of P-gp affect drug absorption and distribution, with effects being predominant at the trough. In particular, among patients taking the higher-dose regimen, we found that the dTT values at the trough, when the *ABCB1 rs4148738* CT genotype was present, were lower than those associated with CC or TT genotypes. This was associated with reduced drug concentrations. Of note, in patients with the *ABCB1 rs4148738 heterozygous CT* genotype, the probability of having dTT values at the trough below the median was 3-fold higher compared to those with CC or TT genotypes. Therefore, it appears that when higher dabigatran doses are used, the CT variant might be protective against drug over-exposure at the trough by acting through reduced drug absorption and increased drug elimination in the bowel. In our cohort, the *ABCB1 rs4148738* heterozygous CT genotype was found in 61 patients (61% of the total population), while the homozygous CC genotype (wild-type) was found in 22 patients and the homozygous TT genotype (mutant trait) in 17 patients. The increase or decrease in dabigatran levels compared to the heterozygotes was not seen in the homozygous genotype, probably due to the small sample size. However, the specific biological mechanism by which *ABCB1 SNP rs4148738* is associated with significant effects on the dabigatran concentrations is a matter of hypotheses. A possible explanation is *ABCB1 rs4148738*, which is a deep intronic variant and is in a linkage disequilibrium with unknown allelic variants, i.e., variants in regulatory regions or variants that influence mRNA splicing, that would modulate the absorption of dabigatran [18]. Interestingly, carrying the *rs4148738* CT genotype was characterized by similar drug concentrations, as well as similar dTT values, in patients receiving 110 and 150 mg dabigatran regimens. Based on our findings, the individual variations in dabigatran pharmacokinetics and pharmacodynamics may be partly explained by genetic polymorphisms. Thus, our investigations may be helpful in predicting the safety and efficacy of dabigatran treatment for each patient based on genotypes and clinical features. It is also plausible that combinations of mutant or heterozygous polymorphisms of *CES1 rs2244613*, *CES1 rs8192935* and *ABCB1 rs4148738* may influence how dabigatran is activated from the prodrug and how it is absorbed in the small intestine. These gene polymorphisms have the potential to influence the pharmacological effect of the drug; however, we found no difference in the ORs between patients with the combination of mutant or heterozygous polymorphisms of *CES1 rs2244613* and *CES1 rs8192935* for the dTT at the trough and peak values below the median, regardless of the dabigatran dose. This hypothesis remains to be confirmed in future and powered studies [16].

Our investigation should be considered in the light of its limitations. In particular, our results need to be confirmed in larger samples. Moreover, they refer to a specific population of Caucasian, Italian patients and it is unknown whether this can be extended to other populations. In addition, this study may be underpowered to detect the effect of SNPs alleles on modest exposure changes. Reported effect sizes could be inflated by the “winner’s curse effect”, a statistical effect resulting in the amplification of SNP-trait association estimates in the sample where these associations are discovered. Finally, a single blood sample at the peak and trough was obtained for each participant; thus, findings were not adjusted for a possible intra-individual variability in dTT values and drug concentrations.

In conclusion, our study, performed in the setting of AF, showed no significant inter-individual variability of anticoagulant effects of dabigatran according to *CES1 SNP rs2244613* and *CES1 SNP rs8192935*. This was regardless of the dabigatran dose. Also, no inter-individual difference in the functional response to the drug was observed based on *ABCB1 SNP rs4148738* in patients receiving the 110 mg dabigatran regimen. Whether the association between the *ABCB1 rs4148738* CT genotype and the reduced anticoagulant effect in patients on the 150 mg dabigatran dose translates into lower bleeding complications and/or reduced thrombotic protection deserves to be addressed in specific, prospective investigations on larger populations with clinical endpoints as outcome measures. However, according to our results, genetic profiling for *ABCB1 SNP rs4148738* and dTT measurement might be empirically indicated in AF patients with recurrent thromboembolic events, despite treatment with the 150 mg dabigatran regimen.

## Figures and Tables

**Table 1:** Main characteristics of the study population. Continuous variables are given as mean ± standard deviation or median (interquartile range), as appropriate. Discrete variables are indicated as *n* (%). AF = Atrial fibrillation; LVEF = Left ventricular ejection fraction; TIA = Transient ischemic attack.

	Overall Population (*n* = 100)	Dabigatran 110 mg (*n* = 43)	Dabigatran 150 mg (*n* = 57)	*p* Value
Age (years)	77 (68–82)	82 (81–86)	70 (67–74)	<0.001
Gender				
Male	63 (63)	23 (53)	40 (70)	0.09
Female	37 (37)	20 (47)	17 (30)	
Weight (Kg)	79 ± 13	74 ± 13	83 ± 13	0.002
Diabetes mellitus	16 (16)	7 (16)	9 (16)	0.88
Systemic hypertension	65 (65)	24 (56)	41 (72)	0.13
Previous stroke/TIA	15 (15)	3 (7)	12 (21)	0.051
Concomitant vascular disease	28 (28)	18 (41)	10 (18)	0.005
Heart failure	14 (14)	5 (12)	9 (16)	0.63
CHA_2_DS_2_VASc score	3 (2–4)	4 (3–4)	3 (2–4)	0.025
HASBLED score	1 (1–2)	1 (1–2)	1 (1–2)	0.08
Type of AF				
Paroxysmal	52 (52)	18 (42)	34 (56)	0.08
Persistent	22 (22)	7 (16)	15 (26)	0.23
Permanent	26 (26)	18 (42)	8 (31)	0.002
Creatinine clearance (mL/min)	71 (59–86)	60 (55–70)	81 (67–90)	<0.001
LVEF (%)	58 (54–62)	60 (54–60)	58 (54–62)	0.88

**Table 2:** Dabigatran plasma concentrations and dTT values in the overall population and according to different dabigatran doses. dTT = diluted thrombin time.

	Overall Population (*n* = 100)	Dabigatran 110 mg (*n* = 43)	Dabigatran 150 mg (*n* = 57)	*p* Value
Dabigatran concentration at trough (ng/mL)	59 (48–87)	69 (49–91)	59 (42–81)	0.37
Dabigatran concentration at peak (ng/mL)	115 (91–171)	113 (100–147)	132 (65–176)	0.94
dTT at trough (ng/mL)	90 (51–132)	90 (54–140)	90 (47–129)	0.94
dTT at peak (ng/mL)	176 (101–232)	174 (87–213)	186 (101–237)	0.53

**Table 3:** Distribution of genetic polymorphisms in the overall population and in the subpopulations receiving different dabigatran doses. GG = guanine/guanine; GT = guanine/thymine; TT = thymine/thymine; AA = adenine/adenine; AG = adenine/guanine; GG = guanine/guanine; CC = cytosine/cytosine; CT = cytosine/thymine; TT = thymine/thymine.

	Overall Population (*n* = 100)	Dabigatran 110 mg (*n* = 43)	Dabigatran 150 mg (*n* = 57)
*CES1 rs2244613 polymorphism*			
GG genotype (wild-type)	5 (5)	-	5 (9)
GT genotype (heterozygous)	35 (35)	17 (39)	18 (31)
TT genotype (mutant trait)	60 (60)	26 (60)	34 (60)
*CES1 rs8192935 polymorphism*			
AA genotype (wild-type)	11 (11)	3 (7)	8 (14)
AG genotype (heterozygous)	43 (43)	21 (49)	22 (39)
GG genotype (mutant trait)	46 (46)	19 (44)	27 (47)
*ABCB1 rs4148738 polymorphism*			
CC genotype (wild-type)	22 (22)	10 (23)	12 (21)
CT genotype (heterozygous)	61 (61)	26 (60)	35 (61)
TT genotype (mutant trait)	17 (17)	7 (16)	10 (18)

**Table 4:** DTT and concentrations at trough and peak for 110 mg and 150 mg dabigatran dose according to different *ABCB1 rs4148738* polymorphisms. CC = cytosine/cytosine; CT = cytosine/thymine; TT = thymine/thymine; dTT = diluted thrombin time.

	Dabigatran 110 mg (*n* = 43)			*p* Value	Dabigatran 150 mg (*n* = 57)			*p* Value
Concentration at trough (ng/mL)	Wild-type homozygosis (CC)	Heterozygosis (CT)	Mutant trait (TT)		Wild-type homozygosis (CC)	Heterozygosis (CT)	Mutant trait (TT)	
	85 (47–98)	58 (48–102)	56 (50–84)	0.76	85 (68–119)	49 (32–59)	78 (62–93)	0.02
Concentration at peak (ng/mL)	Wild-type homozygosis (CC)	Heterozygosis (CT)	Mutant trait (TT)		Wild-type homozygosis (CC)	Heterozygosis (CT)	Mutant trait (TT)	
	108 (102–132)	126 (99–209)	109 (91–115)	0.59	189 (129–232)	132 (55–175)	90 (65–117)	0.24
dTT at trough (ng/mL)	Wild-type homozygosis (CC)	Heterozygosis (CT)	Mutant trait (TT)		Wild-type homozygosis (CC)	Heterozygosis (CT)	Mutant trait (TT)	
	114 (69–151)	74 (42–128)	93 (88–145)	0.23	127 (85–147)	77 (44–111)	110 (47–159)	0.048
dTT at peak (ng/mL)	Wild-type homozygosis (CC)	Heterozygosis (CT)	Mutant trait (TT)		Wild-type homozygosis (CC)	Heterozygosis (CT)	Mutant trait (TT)	
	172 (153–188)	172 (70–213)	187 (150–241)	0.70	214 (159–295)	163 (101–237)	178 (86–236)	0.28

## Data Availability

Our research obtained informed consent from everyone who met the inclusion criteria. The data underlying this article are available in the article and its online Supplementary Materials.

## Supplementary Materials

The following supporting information can be downloaded at: https://www.mdpi.com/article/10.3390/jcm13092545/s1, Supplementary Table S1: dTT and concentrations at trough and at peak for dabigatran 110 mg and 150 mg dose according to different *CES1 rs2244613* polymorphisms; Supplementary Table S2: dTT and concentrations at trough and at peak for dabigatran 110 mg and 150 mg dose according to different *CES1 rs8192935* polymorphisms.
