# Supplementary material for: Role of CES1 and ABCB1 Genetic Polymorphisms on Functional Response to Dabigatran in Patients with Atrial Fibrillation
Source: J Clin Med. 2024 Apr 26;13(9):2545. doi: 10.3390/jcm13092545 (PMC11084678; doi:10.3390/jcm13092545)
Supplement: Supplementary file 1 [file jcm-13-02545-s001.zip › jcm-2948505-supplementary.pdf]

	Dabigatran 110 mg (n=43)			P value	Dabigatran 150 mg (n=57)			P value
Concentration at trough (ng/mL)	Wild-type homozygosis (GG)	Heterozygosis (GT)	Mutant trait (TT)		Wild-type homozygosis (GG)	Heterozygosis (GT)	Mutant trait (TT)	
	-	84 (43-92)	58 (50-91)	0.69	83 (82-84)	59 (58-69)	58 (42-76)	0.51
Concentration at peak (ng/mL)	Wild-type homozygosis (GG)	Heterozygosis (GT)	Mutant trait (TT)		Wild-type homozygosis (GG)	Heterozygosis (GT)	Mutant trait (TT)	
	-	109 (89-156)	115 (101-144)	0.32	93 (92-94)	146 (107-186)	132 (64-175)	0.72
dTT at trough (ng/mL)	Wild-type homozygosis (GG)	Heterozygosis (GT)	Mutant trait (TT)		Wild-type homozygosis (GG)	Heterozygosis (GT)	Mutant trait (TT)	
	-	90 (42-145)	90 (55-139)	0.72	129 (96-135)	77 (43-127)	92 (57-125)	0.47
dTT at peak (ng/mL)	Wild-type homozygosis (GG)	Heterozygosis (GT)	Mutant trait (TT)		Wild-type homozygosis (GG)	Heterozygosis (GT)	Mutant trait (TT)	
	-	161 (75-207)	176 (148-213)	0.59	185 (154-224)	168 (135-238)	190 (100-237)	0.92

Supplementary Table S1. dTT and concentrations at trough and at peak for dabigatran 110 mg and 150 mg dose according to different *CES1 rs22444613* polymorphisms. GG: guanine-guanine; GT: guanine-thymine; TT: thymine-thymine; dTT: diluted thrombin time

	Dabigatran 110 mg (n=43)			P value	Dabigatran 150 mg (n=57)			P value
Concentration at trough (ng/mL)	Wild-type homozygosis (AA)	Heterozygosis (AG)	Mutant trait (GG)		Wild-type homozygosis (AA)	Heterozygosis (AG)	Mutant trait (GG)	
	92 (91-93)	84 (48-192)	51 (50-58)	0.42	71 (58-83)	64 (31-81)	58 (42-76)	0.79
Concentration at peak (ng/mL)	Wild-type homozygosis (AA)	Heterozygosis (AG)	Mutant trait (GG)		Wild-type homozygosis (AA)	Heterozygosis (AG)	Mutant trait (GG)	
	109 (108-110)	114 (91-156)	115 (103-132)	0.86	100 (93-106)	160 (31-186)	132 (64-176)	0.74
dTT at trough (ng/mL)	Wild-type homozygosis (AA)	Heterozygosis (AG)	Mutant trait (GG)		Wild-type homozygosis (AA)	Heterozygosis (AG)	Mutant trait (GG)	
	106 (42-160)	106 (59-143)	74 (42-95)	0.42	94 (58-135)	84 (42-113)	98 (58-139)	0.40
dTT at peak (ng/mL)	Wild-type homozygosis (AA)	Heterozygosis (AG)	Mutant trait (GG)		Wild-type homozygosis (AA)	Heterozygosis (AG)	Mutant trait (GG)	
	206 (75-214)	177 (139-232)	168 (72-204)	0.70	182 (136-258)	168 (101-215)	191 (100-240)	0.56

Supplementary Table S2. dTT and concentrations at trough and at peak for dabigatran 110 mg and 150 mg dose according to different *CES1 rs8192935* polymorphisms. AA: adenine-adenine; AG: adenine-guanine; GG: guanine-guanine. dTT= diluted thrombin time
